# Dental Adaptation Strategies for Children with Autism Spectrum Disorder—A Systematic Review of Randomized Trials

**DOI:** 10.3390/jcm13237144

**Published:** 2024-11-26

**Authors:** Magdalena Prynda, Agnieszka Anna Pawlik, Wojciech Niemczyk, Rafał Wiench

**Affiliations:** 1 Orthodontic Specialist, M-Dent Center for Esthetic Dentistry and Implantology, 34a/7 Sienkiewicza St., 50-335 Wrocław, Poland; 2 Specialist DentalClinic dr n.med.Agnieszka Anna Pawlik ul, Strumieńskiego 12/4, 41-400 Mysłowice, Poland; pawlik.paro@gmail.com; 3 Department of Periodontal Diseases and Oral Mucosa Diseases, Faculty of Medical Sciences in Zabrze, Medical University of Silesia, Pl. Traugutta 2, 41-800 Zabrze, Poland

**Keywords:** autism spectrum disorder, behavior management, oral health

## Abstract

**Background:** Children with Autism Spectrum Disorder (ASD) often struggle with dental care due to sensory sensitivities and behavioral issues, increasing their risk for oral health problems. Adaptation strategies such as visual aids, video modeling, and sensory-adapted environments aim to improve their dental experiences. **Methods:** A systematic review of randomized controlled trials (RCTs) was conducted according to PRISMA 2020 guidelines using the PubMed, Scopus, Embase, and Cochrane databases. Of the 1072 records screened, nine RCTs were included in the analysis. Studies included children with ASD under 18 years and compared dental adaptation techniques with traditional care. The risk of bias and study quality were assessed. The quality of evidence for the results was determined using the GRADE tool. **Results:** Nine RCTs with sample sizes ranging from 25 to 138 participants showed significant improvements in oral hygiene, reduced anxiety, and increased cooperation. Video modeling and sensory-adapted environments were particularly effective in lowering distress during dental visits. **Conclusions:** Dental adaptation strategies, especially video modeling and sensory-adapted environments, effectively improve oral health outcomes and reduce anxiety in children with ASD. More research is needed to explore the long-term effects and include children with severe ASD.

## 1. Introduction

Autism spectrum disorder (ASD), also referred to as autism, is a prevalent neurodevelopmental disorder with a strong genetic component and a wide range of manifestations. It is characterized by underlying cognitive features and frequently co-occurs with other conditions. The core symptom domains of ASD can be broadly classified into two categories: social communication impairments and restricted and repetitive patterns of behavior and interests [1,2,3]. It is a common occurrence for individuals with ASD to report atypical responses to sensory stimuli [4,5,6]. Overreactivity to sound and underreactivity to pain were reported by more than 40% of children with ASD. The prevalence of sensory abnormalities was found to be higher among individuals with more severe autistic traits compared to those with less severe autistic traits [4]. Furthermore, sensory sensitivities in children with ASD have been linked to behavioral challenges in dental settings [7]. The global prevalence of autism is estimated to be approximately 1%, although this figure is thought to be higher in countries with a high income per capita [8,9,10]. Patients with ASD do not exhibit any distinctive dental characteristics in the soft or hard intraoral or perioral tissues [11,12,13]. However, patients with ASD have greater dental needs compared to individuals without ASD [14]. A systematic review and meta-analysis revealed a high prevalence of dental caries and periodontal disease among children and young adults with ASD [15,16,17,18,19,20]. The Caries Risk Assessment Tool as adapted by the American Academy of Pediatric Dentistry indicates that children with ASD are at a significantly elevated risk for dental caries. This is attributed to a number of factors, including a proclivity for carious food, the tendency to pouch food due to poor masticatory ability, inadequate maintenance of oral hygiene, and the need for assistance with tooth brushing [21]. Moreover, other factors such as delayed eruption of the teeth, oral trauma and injury (including biting lips and picking at the gums), bruxism (or teeth grinding), non-nutritive chewing (eating objects), and tongue thrusting have all been identified as potential contributors to the oral health issues commonly associated with ASD. The elevated risk of compromised oral health in individuals with ASD may be attributed to specific pharmacological agents, certain behavioral patterns, and challenges in adhering to daily hygiene routines [22,23]. The presence of poor oral health significantly impacts the overall quality of life in individuals with ASD. Consequently, it is of the utmost importance to incorporate regular dental assessment and treatment into the therapeutic protocols for patients with ASD [24]. Children with ASD frequently exhibit a lack of tolerance for dental interventions, often due to fear of sights and sounds in the dental setting, which is known as perceptual hypersensitivity [25,26]. Children with ASD tend to be highly dependent on routine. Consequently, activities outside of their routine, such as a dental appointment, can cause significant distress to the child and dental office staff [27]. The duration of the visit should be brief, and any sensory stimuli should be kept to a minimum. A combination of desensitization, symbolic remodeling, and reinforcement can also facilitate the dental examination of autistic patients [28]. In numerous instances, the dental treatment of patients necessitated the utilization of sophisticated behavioral guidance techniques, including sedation and general anesthesia [2]. This systematic review assesses the efficacy of dental adaptation strategies for children with ASD by synthesizing evidence from randomized controlled trials published in the last five years and provides dental practitioners and researchers with a comprehensive understanding of the most effective approaches to enhance dental care experiences and outcomes for children with ASD. In addition, it addresses critical challenges in dental practice and offers guidance on future research directions. In light of the increasing prevalence of ASD and the urgent need for efficacious dental care adaptations, this review not only assesses the immediate outcomes of these interventions but also considers their broader implications for practice and future research. In this systematic review, the authors conducted a qualitative examination of the null hypothesis that dental adaptation strategies, including visual aids, video modeling, and sensory-adapted environments, do not significantly improve oral health outcomes, reduce anxiety, or increase cooperation in children with ASD compared to traditional dental care approaches. Even though a meta-analysis was not feasible due to the heterogeneity across studies, the authors evaluated the individual study results in order to assess the patterns that could either support or challenge this hypothesis.

## 2. Materials and Methods

### 2.1. Focused Question

A systematic review was conducted in accordance with the PICO framework [29], with the aim of ensuring the highest standards of rigor and transparency. In children with autism spectrum disorder (Population), are dental adaptation strategies such as visual aids or video modeling (Intervention) more effective than traditional dental care methods (Comparison) in improving oral hygiene, reducing dental anxiety, and enhancing cooperation during dental visits (Outcome)?

### 2.2. Search Strategy

This systematic review was conducted in accordance with the Preferred Reporting Items for Systematic Reviews and Meta-Analyses (PRISMA 2020) guidelines [30]. An electronic literature search was conducted using the PubMed, Scopus, Embase, and Cochrane databases. Syntaxes composed of keywords and Boolean operators are shown separately for each of the databases used in Table 1 below. In addition, it shows the number of results for the given syntaxes and what search constraints were used. In addition, the authors performed an additional snowball search of articles cited by articles assessed for eligibility. To avoid missing key articles, three authors (M.P., W.N., R.W.) performed searches using the same search parameters, in the same databases. All authors then jointly decided on the inclusion of the retrieved articles, with 100% concordance.

### 2.3. Selection of Studies

The objective of this systematic review was to assess the efficacy of dental adaptation strategies, such as the use of visual aids and video modeling, in comparison to traditional dental care methods for children with autism spectrum disorder. This review aimed to evaluate improvements in oral hygiene, reductions in dental anxiety, and enhanced cooperation during dental visits. Table 2 presents the detailed inclusion and exclusion criteria for the systematic review.

### 2.4. Data Extraction

#### 2.4.1. Risk of Bias in Individual Studies

In the initial phase of the study selection process, each reviewer was encouraged to assess titles and abstracts individually, to mitigate potential biases in the evaluation process. In order to quantify the level of inter-reviewer agreement, the researchers employed the use of Cohen’s k test for every database used [31]. Any discrepancies regarding the inclusion or exclusion of a study in the review were discussed by the authors until a consensus was reached (Cohen’s k test result between 0.81 and 1.00).

#### 2.4.2. Quality Assessment

Two reviewers (M.P. and W.N.) undertook independent screenings of the included studies with a view of assessing their quality. In order to evaluate the study design, implementation, and analysis, the following criteria were taken into consideration: whether patients were randomly assigned to study groups; whether there was a blinding component; whether the group sizes of the study groups were calculated in advance before the study; and whether, in the process of randomization and loss of patients to follow-up, overall, the final numbers in both groups were balanced (with an acceptable error of 10%). A very important criterion, which at the same time was a condition for the study to be accepted for analysis, was the patient’s confirmed diagnosis of ASD. Another criterion was the inclusion of patients with all three degrees of ASD (mild, moderate, and severe). The authors also assessed whether the evaluation of the results was performed objectively and whether appropriate statistical tests were used to process the results. The study received 1 point for each of the favorable factors (indicated as a green circle with +). If a criterion was not met, the study received no point (indicated as a red circle with an X). The maximum number of points a study could receive was 9. The level of risk of bias associated with a given study was contingent upon its score. A score between 0 and 3 points would indicate a high risk of bias and, consequently, the study would be excluded from the narrative synthesis. A score of 4 to 6 indicates a medium risk of bias, while studies with scores between 7 and 9 are deemed to have a low risk of bias.

### 2.5. Evidence Quality Assessment

A systematic assessment of the synthesis and quality of the evidence for each outcome was conducted in accordance with the Grading of Recommendations, Assessment, Development, and Evaluation (GRADE) approach [32]. The quality of the evidence was categorized into four tiers: high, moderate, low, and very low. Given the subjective nature of GRADE parameter evaluation, the three authors (M.P., W.N., R.W.) conducted a separate evaluation, with a consensus reached through discussion and the use of Cohen’s k test.

## 3. Results

### 3.1. Study Selection

**Figure 1:**
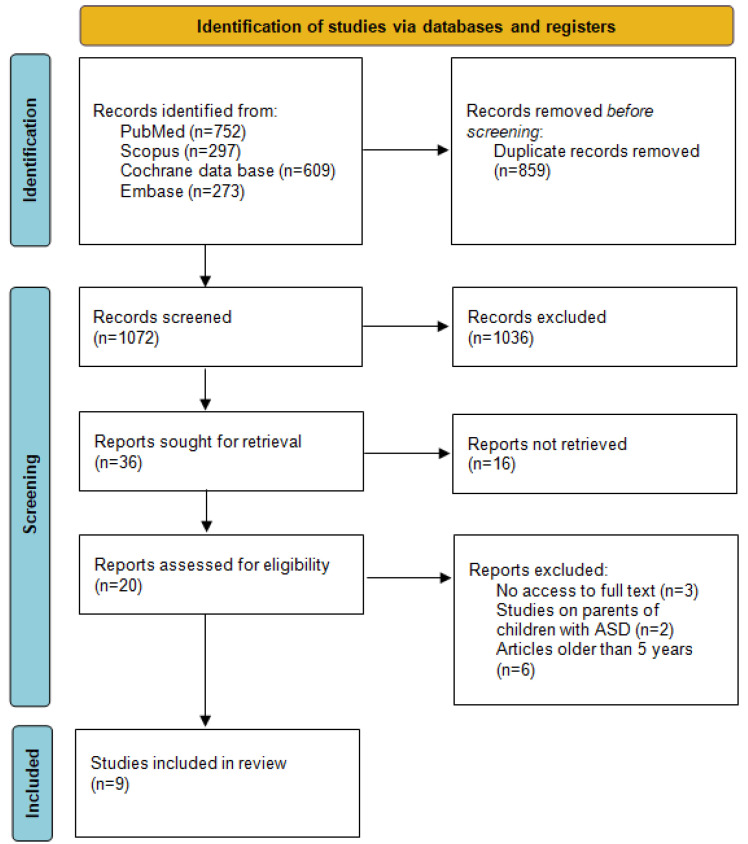
PRISMA 2020 flow diagram.

In the initial phase of the process, all instances of repetition were removed from the four databases, leaving a total of 1072 articles for further examination. Subsequently, 45 articles were selected for retrieval. Of the identified articles, 29 were assessed for eligibility. Of these, six were excluded due to the date of publication preceding 2019 [33,34,35,36,37,38]. The analysis was thus conducted on nine articles. A flowchart of the research approach according to the PRISMA 2020 statement [30] is shown in Figure 1.

### 3.2. Risk of Bias Across Studies

In accordance with the guidelines set forth in the Cochrane Handbook for Systematic Reviews of Interventions [39], an assessment of the risk of bias was conducted for each of the included articles. Of the nine articles subjected to assessment, none were excluded on the grounds of a high risk of bias. The lowest score was 6, indicating a moderate risk of bias for two studies [40,41]. The remaining seven studies were deemed to exhibit a low risk of bias. Four studies achieved an overall score of 8 [42,43,44,45], while three studies obtained a score of 7 out of a total of 9 [46,47,48]. The sole criterion that was met by only one study [48] was the inclusion of patients with all degrees of ASD. Table 3 below presents the detailed risk of bias scores for the included studies.

### 3.3. General Characteristics of the Included Studies

The general characteristics of the nine studies included in this review are summarized in Table 4. One of the criteria for including articles was publication within the last 5 years. However, the oldest articles included are from 2022. The studies were conducted across various countries, including Saudi Arabia [42,43,44], Italy [46], Brazil [45], the USA [40,48], Iran [41], and Egypt [47]. The sample sizes varied, ranging from 25 to 138 participants, with most studies including both male and female children, except for Piraneh et al. [41], which only included male participants. In five studies [42,43,44,45,48], the upper age limit was 12 years. Only Shalabi et al. did not provide criteria as to the age of the patients included in the study, but it is known that they were children. All three studies by Aljubour et al. [42,43,44] were conducted on the same patients at the same time. The results were separated into three separate publications.

### 3.4. Study Outcomes

#### 3.4.1. Oral Hygiene

Several studies reported significant improvements in oral hygiene following the implementation of specific dental interventions. Aljubour et al. (2022) [42] found that the use of culturally adapted dental visual aids (DVA) resulted in significant improvements in the oral hygiene status of children with ASD, with the test group showing greater improvements than the control group (*p* = 0.030). Similarly, Gandhi et al. (2024) [40] and Piraneh et al. (2023) [41] observed significant improvements in plaque and gingival scores and oral hygiene status in children who received interventions such as video modeling (VM) or tooth brushing social stories (TSS). Piraneh’s study highlighted that video modeling was particularly effective in improving Oral Hygiene Index-Simplified (OHI-S) scores compared to traditional social stories (*p* < 0.001) [41]. Several studies included follow-up periods to assess the maintenance of intervention effects. Shalabi et al. (2022) reported that video modeling was more effective than the Picture Exchange Communication System (PECS) in reducing OHI-S scores over 12 months, with significant improvements observed at 3-, 6-, and 12-months post-intervention (*p* < 0.001). This evidence highlights the long-term benefits of video modeling in sustaining improved oral health behaviors in children with ASD [47]. There was a high quality of evidence according to the GRADE rating.

#### 3.4.2. Anxiety and Stress Reduction

A reduction in dental anxiety was identified as a key outcome in multiple studies. Aljubour et al. (2023) [43] demonstrated that the use of culturally adapted visual aids led to a significant decrease in anxiety levels in children with ASD during dental visits compared to the control group. The utilization of visual aids effectively addressed the unique communication and understanding challenges faced by autistic children, thereby making dental visits less distressing. Stein Duker et al. (2023) evaluated the physiological stress and behavioral distress experienced during dental visits, comparing sensory-adapted dental environments (SADE) with regular dental environments (RDE). The children in the SADE group exhibited significantly lower physiological stress (*p* < 0.001) and reduced behavioral distress, as indicated by the frequency and duration of distress behaviors during dental care, as observed via video coding. These findings indicate that sensory adaptations, which address sensory sensitivities common in children with ASD, can markedly enhance their overall experience during dental visits [48]. There was a moderate quality of evidence according to the GRADE rating.

#### 3.4.3. Behavioral Improvement

It was also demonstrated that behavioral interventions were instrumental in enhancing cooperation during dental visits. The studies conducted by Aljubour et al. (2024) [44] and Cirio et al. (2022) [46] demonstrated that children who were exposed to behavioral techniques, such as video or photo modeling, exhibited superior behavior and cooperation compared to the control groups. The 2024 study by Aljubour revealed a notable enhancement in behavioral patterns within the test group (*p* < 0.001) [44]. Conversely, Cirio’s study indicated that children in the video group demonstrated a greater inclination to complete the initial stages of the dental examination process, although this discrepancy reached statistical significance only for the preliminary steps (*p* = 0.04) [46]. The efficiency of dental care was also evaluated as a further outcome measure. In a study conducted by Da Silva Moro et al. (2024), it was observed that children who watched a video prior to dental consultations required fewer visits to complete the necessary dental procedures in comparison to the control group (1.5 vs. 2 visits, *p* ≤ 0.05). This underscores the potential of video modeling techniques to foster cooperation and reduce the overall time required for dental treatment in children with ASD [45]. There was a high quality of evidence according to the GRADE rating. The details of all those included in the analysis are shown in Table 5.

### 3.5. GRADE Ratings

The findings of four studies, encompassing a total of 272 patients, were related to oral health. Anxiety and stress were addressed in only two studies, which included a total of 88 patients. The majority of studies focused on behavioral improvement, although this was not the focus of the largest number of patients, which included a total of 237 of them. As all of the included studies were randomized trials, they all started the assessment with a high quality of evidence.

Inconsistency was assessed according to the GRADE Handbook on the basis of heterogeneity values ($I^{2}$): <40% may be low, 30–60% may be moderate, 50–90% may be substantial, 75–100% may be considerable. The quality of the evidence is as follows: There is a high quality of evidence that oral health and behavior may be improved—the research provides a high level of indication as to the likely effect. The evidence pertaining to anxiety and stress is of moderate quality. This indicates that the research provides a substantial indication of the probable effect. The likelihood of a notable discrepancy in the observed effect is moderate. The overall quality and summary of evidence using the GRADE approach are presented in Table 6.

## 4. Discussion

### 4.1. General Interpretation and Comparison

The findings of the included studies largely contradict the null hypothesis, which suggests that dental adaptation strategies have no significant impact on outcomes for children with ASD. The majority of studies reported meaningful improvements in anxiety reduction, oral hygiene, and cooperation levels associated with these adaptations, indicating that these strategies may offer clinical benefits. However, due to methodological variability and the absence of pooled statistical analysis, these findings should be interpreted with caution, and further research is required to confirm their generalizability and long-term effectiveness. The included studies evaluated the effectiveness of various dental interventions aimed at improving oral health outcomes and reducing dental anxiety in children with ASD. Key outcomes assessed in these studies included improvements in oral hygiene, reductions in dental anxiety and behavioral distress, and increased cooperation during dental visits. Four of the included studies focused on the assessment of hygiene indicators in the children studied. All of them showed significant improvements in the parameters studied [40,41,42,47]. Three of them studied VM [40,41,47], while one of the studies was based on DVA [42]. It should also be kept in mind that in the study conducted by Aljubour et al., only 15.6% of children brushed their teeth independently. In other studies, these figures were not reported [42]. VM represents a highly efficacious strategy for the development of a range of competencies in individuals with ASD, including social, communication, and daily living skills. The VM approach entails the presentation of a video in which a model exemplifies a target behavior or a specific task, after which the participant is required to demonstrate the skills observed in the video [49,50]. Similar results regarding VM in children with ASD were obtained by Popple et al. in their randomized trial, where they noted a significant improvement in the clinical subjects tested [27]. Isong et al. showed a significantly greater reduction in anxiety in patients with ASD in whom VN was used compared to children in other groups, with both the control and intervention groups relying on glasses displaying the children’s favorite cartoons [37]. Aljubour et al. conducted a single study, which was published in three separate works on the topic of DVA. The results obtained were significantly superior to those of the control group. A systematic review also conducted by Aljubour et al. (2021) revealed that the utilization of dental visual aids is an efficacious method for enhancing the cooperation of children with ASD during dental treatment, while also serving to exemplify appropriate behavior and mitigate anxiety [51]. A similar method of adaptation, only in the form of a book to prepare children for a visit, was used by Murshid in his study. The results of his study showed that 47.5% of children with ASD behaved positively during dental treatment, and 37.5% had a positive effect on children’s behavior as rated by their parents [52]. The positive effect of visual aids on children with ASD was also observed in the study by Cagetti et al. [53]. There are also studies using a sensory-adapted dental environment (SADE) that confirm improvements in children’s oral hygiene [25,38,54,55]. However, it should be noted that the effects of SADE extend beyond mere oral hygiene in children with ASD. Ismail et al. conducted a systematic review of four studies evaluating the efficacy of SADE on children with special needs who had undergone dental treatment. The studies analyzed demonstrated that children with special needs who received dental treatment in SADE exhibited notable improvements in physiological changes, behavior, pain, and sensory discomfort [56]. A randomized study by Cermak et al. demonstrated a statistically significant reduction in electrodermal activity in the SADE group when compared to the RDE group. The effect size of the comparison between SADE and RDE was 0.23 ASD/0.29 non-ASD [38]. Similar results regarding SADE were also reached by Stein Duker et al., whose study was included in the analysis [48]. It is important for children with ASD to maintain proper oral hygiene, as children with ASD have a significantly higher risk of dental caries with the same dietary habits as neuromuscular children. In the study of 58 Pakistani children with ASD matched with 27 siblings without ASD, despite similar diets and sugar intake, the children with ASD were more likely to have dental decay (50 percent) than the siblings without ASD (22.2 percent), with 24 percent of the ASD children having dental decay compared to 14 percent of the siblings without ASD [14]. A further issue is that children with ASD not only express a preference for sweet snacks but also demonstrate selective diets or “picky” eating habits. These behaviors have recently been associated with an aberrant sensory experience, such as heightened reactivity to food tastes and textures [57,58]. Shalabi et al. [47] were the sole authors to conduct a follow-up period of 12 months. Moreover, they were the sole researchers to investigate the use of PECS. Although it demonstrated inferiority compared to VM, PECS exerted a notable impact on the efficacy of the parameters under investigation. Al-Batayneh et al. also observed comparable outcomes with regard to the utilization of PECS on gingival well-being [59]. In their pilot study, Zink et al. demonstrated that the use of PECS facilitated patient–professional communication during preventive procedures in individuals with ASD, including those with previous dental experience [60]. In 2018, however, Zink et al. conducted a randomized trial comparing PECS to a proprietary phone app. The results of this study indicated that the app was more effective than the PECS for dentist–patient communication, resulting in a reduction in the number of appointments required for preventive dental care and clinical examinations [33]. In previous years, the authors also employed the Tell–Show–Do (TSD) method with children diagnosed with ASD with notable success. The utilization of this method has been observed to enhance the level of cooperation exhibited by children during dental treatment, facilitate the completion of a greater number of steps during the final visit, reduce the time required to achieve the desired level of cooperation, and mitigate the occurrence of behavioral anxiety [35,36,61,62]. The included studies demonstrated that dental adaptation interventions, particularly culturally adapted visual aids, video modeling, and sensory-adapted environments, were significantly effective in improving oral hygiene, reducing anxiety and distress, and enhancing cooperation during dental visits in children with ASD. These strategies also contributed to more efficient dental care, with a reduction in the number of required visits and the maintenance of long-term improvements.

### 4.2. Limitations

This systematic review is not without limitations. The data synthesis was performed narratively due to the different nature of the included studies. Although the authors tried to select articles of the highest quality by including only randomized trials, not all studies had a low risk of bias. Another notable limitation is that all but one of the authors of the included studies chose to exclude children with severe ASD. This may distort the results by relating them to all children with ASD. Most of the studies also did not have the option of blinding due to their methodology, which also increases the risk of bias. It is also noteworthy that multicenter studies were not included in the analysis due to their lack. The risk of bias may also have been influenced by the age of the children selected for the study. This is because children over the age of 12 may also be affected by hormonal changes that may lead to disobedience and less willingness to cooperate [63,64]. Also of note is the relatively short follow-up in most of the included studies. Studies with longer follow-ups would be useful to assess the durability of the adaptations and whether they have a long-term impact on oral hygiene in children with ASD. A further limitation of this review is that some of the studies included an absence of dental history as a criterion for inclusion [42,43,44]. This is a reasonable approach, as it avoids introducing bias into the results by including patients who may have experienced dental trauma. In contrast, the study by Gandhi et al. [40] employed dental history and parental issues related to their children with ASD’s acceptance of oral hygiene at home as inclusion criteria. The absence of gender balance among the children studied represents a further limitation. It should be noted that a greater number of boys were surveyed than girls. Nevertheless, this is substantiated by the fact that males are more than four and a half times more prone to developing ASD than females [65].

### 4.3. Strengths of the Study

Despite its limitations, this study had significant advantages, including the assessment of the risk of bias in the articles. The search was conducted independently by three authors using a comprehensive set of search criteria to minimize the risk of not identifying significant experiments. The inclusion and exclusion criteria were applied consistently, resulting in a limited number of articles being reviewed. In addition to the narrative synthesis of the data, an assessment of the quality of the evidence was conducted using the GRADE tool.

### 4.4. Implications of the Results for Practice, Policy, and Future Research

The results of the included studies may contribute to algorithms for the management of young patients with ASD. Culturally and regionally adapted educational materials may prove helpful in adapting children and guiding their proper oral hygiene habits. Future studies should also include patients with severe ASD, as these are the children who may need the most help. Multicenter studies may also reduce the risk of bias and lead to more substantive results. It is also necessary to consider a longer follow-up period to test the durability of the adaptation.

## 5. Conclusions

This systematic review demonstrates that dental adaptation strategies, including culturally adapted visual aids, video modeling, and sensory-adapted environments, significantly improve oral health outcomes and reduce anxiety in children with ASD. These interventions effectively address sensory sensitivities and behavioral challenges, promoting enhanced cooperation during dental visits. While short-term outcomes are promising, there are limited data on the long-term benefits and effectiveness for children with severe ASD. Future research should involve longer follow-up periods and include multicenter studies to verify the sustainability of these adaptations. Ultimately, implementing these approaches broadly in dental practice may reduce the need for sedation or general anesthesia, thereby enhancing the quality of care for children with ASD.

## Figures and Tables

**Table 1:** Presentation of syntaxes, search limits, and number of results for given parameters.

Source	Search Term	Filters	Number of Results
Medline via PubMed	(“autism” OR “autism disorder” OR “autistic disorder” OR “Asperger syndrome” OR “Rett syndrome” OR “autism” OR “autism spectrum disorders” OR “Asperger” OR “developmental disorder” OR “pervasive child development disorders” OR “pervasive developmental disorder” OR “early infantile autism” OR “Kanner syndrome” OR “infantile autism” OR “ASD”) AND (“dentophobia” OR “dental fear” OR “dental anxiety” OR “dental anxieties” OR “adaptation” OR “modeling” OR “dental phobia” OR “odontophobia”)	Randomized Controlled Trials	752
Web of Science Scopus	(“autism” OR “autism disorder” OR “autistic disorder” OR “Asperger syndrome” OR “Rett syndrome” OR “autism spectrum disorders” OR “Asperger” OR “developmental disorder” OR “pervasive child development disorders” OR “pervasive developmental disorder” OR “early infantile autism” OR “Kanner syndrome” OR “infantile autism” OR “ASD”) AND (“dentophobia” OR “dental fear” OR “dental anxiety” OR “dental anxieties” OR “adaptation” OR “modeling” OR “dental phobia” OR “odontophobia”)	Randomized Controlled Trials	297
Cochrane database	(“autism” OR “autism disorder” OR “autistic disorder” OR “Asperger syndrome” OR “Rett syndrome” OR “autism” OR “autism spectrum disorders” OR “Asperger” OR “developmental disorder” OR “ASD”) AND (“dentophobia” OR “dental fear” OR “dental anxiety” OR “dental anxieties” OR “adaptation” OR “modeling” OR “dental phobia” OR “odontophobia”)	Trials	609
Embase	(‘autism’ OR ‘autism disorder’ OR ‘autistic disorder’ OR ‘asperger syndrome’ OR ‘rett syndrome’ OR ‘autism spectrum disorders’ OR ‘asperger’ OR ‘developmental disorder’ OR ‘pervasive child development disorders’ OR ‘pervasive developmental disorder’ OR ‘early infantile autism’ OR ‘kanner syndrome’ OR ‘infantile autism’ OR ‘asd’) AND (‘dentophobia’ OR ‘dental fear’ OR ‘dental anxiety’ OR ‘dental anxieties’ OR ‘adaptation’ OR ‘modeling’ OR ‘dental phobia’ OR ‘odontophobia’)	Randomized Controlled Trials	273

**Table 2:** Selection criteria for papers included in the systematic review.

Inclusion criteria: Full text available English language Randomized trials Patients aged <18 years Low or moderate risk of bias Autism spectrum disorder diagnosis confirmed Published in last 5 years	Exclusion criteria: Case reports/Case series Narrative reviews Systematic reviews Meta-analysis Non-English language publications Letters to editor Conference papers Non-peer-reviewed literature Gray literature Studies based on parents’ education

**Table 3:** The results of the quality assessment and risk of bias across the studies.

Study									
Criteria	Aljubour et al. (2022) [42]	Aljubour et al. (2023) [43]	Aljubour et al. (2024) [44]	Cirio et al. (2022) [46]	Da Silva Moro et al. (2024) [45]	Gandhi et al. (2024) [40]	Piraneh et al. (2023) [41]	Shalabi et al. (2022) [47]	Stein Duker et al. (2023) [48]
Random allocation	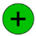	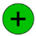	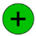	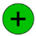	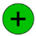	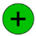	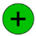	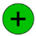	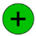
Study was blinded	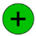	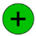	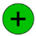	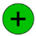	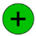	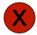	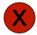	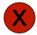	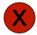
Calculated study group	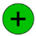	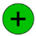	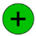	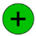	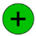	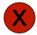	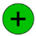	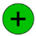	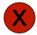
Balanced study groups (+/−10%)	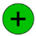	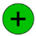	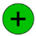	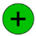	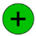	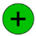	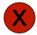	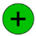	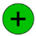
Inclusion/exclusion criteria clearly defined	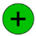	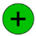	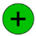	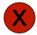	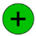	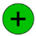	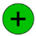	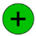	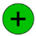
Autism spectrum disorder diagnosis confirmed	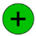	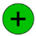	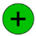	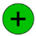	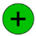	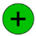	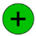	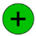	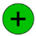
Patients not excluded due to ASD severity	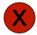	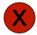	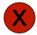	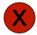	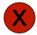	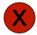	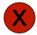	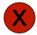	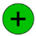
Primary clinical outcome(s) measured objectively	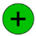	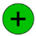	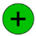	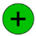	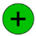	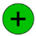	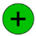	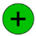	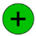
Adequate statistical analysis	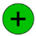	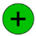	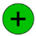	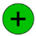	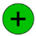	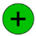	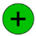	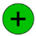	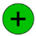
Total	8	8	8	7	8	6	6	7	7
Risk of bias	Low	Low	Low	Low	Low	Moderate	Moderate	Low	Low

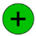
—Indicates that the article has met the criterion; 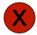
—Indicates that the article has not met the criterion.

**Table 4:** Characteristics of study designs and patients by study.

Author/Year	Country	Study Desing	Sample Size Calculation	Patients	Sex		Age (Years)	
					Female	Male	Mean (±SD)	Range
Aljubour et al. (2022) [42]	Saudi Arabia	BRCT	Yes	64	21	43	8.2	6–12
Aljubour et al. (2023) [43]	Saudi Arabia	BRCT	Yes	64	21	43	8.2	6–12
Aljubour et al. (2024) [44]	Saudi Arabia	BRCT	Yes	64	21	43	8.2	6–12
Cirio et al. (2022) [46]	Italy	BRCT	Yes	84	12	72	7.54 ± 2.42	3–15
Da Silva Moro et al. (2024) [45]	Brazil	BRCT	Yes	40	4	36	7.12 ± 2.24	4–12
Gandhi et al. (2024) [40]	USA	RCT	No	25	2	23	9.5 ± 3.1	4–17
Piraneh et al. (2023) [41]	Iran	RCT	Yes	133	0	133	11.57 ± 2.29	7–15
Shalabi et al. (2022) [47]	Egypt	RCT	Yes	50	15	35	8.6±1.1	<18
Stein Duker et al. (2023) [48]	USA	RCT	No	138	24	114	9.16 ±1.99	6–12

BRCT—Blinded Randomized Controlled Trial, RCT—Randomized Controlled Trial.

**Table 5:** Detailed characteristics of the studies included in this review.

Author/Year	Treatment Groups	Number of Dental Visits	Evaluation	Main Results	Follow-Up Period
Aljubour et al. (2022) [42]	1. Control group—regular DVA 2. Test group—culturally adapted DVA	2	Plaque Index Scores	There was a notable enhancement in the oral health status of both groups following the utilization of the dental visual aids (*p* < 0.001, *p* < 0.001), respectively. A significant improvement in OH status was observed in the test group in comparison to the control group (*p* = 0.030). The two dental visual aids demonstrated efficacy in enhancing oral health status in children with autism spectrum disorder.	4 weeks
Aljubour et al. (2023) [43]	1. Control group—regular DVA 2. Test group—culturally adapted DVA	2	Anxiety Scale for Children with Autism Spectrum Disorder	A statistically significant reduction in anxiety levels was observed in the test group compared to the control group (*p* < 0.001). The culturally adapted dental visual aids were demonstrated to effectively reduce anxiety levels in children with autism spectrum disorder during dental visits.	4 weeks
Aljubour et al. (2024) [44]	1. Control group—regular DVA 2. Test group—culturally adapted DVA	2	Observational Scale of Behavioral Distress	There was a notable alteration in behavioral patterns among the test group (*p* < 0.001), whereas no statistically significant change was observed in the control group (*p* = 0.077). Concerning behavioral patterns, the experimental group demonstrated a significantly superior performance compared to the control group (*p* < 0.001).	4 weeks
Cirio et al. (2022) [46]	1. Video Group 2. Photo Group	1	Frankl Behavioral Scale; Evaluation of the steps needed to complete first dental examination (1–8)	The video group demonstrated a greater number of steps achieved; however, the comparison between groups was statistically significant only for the preliminary steps (*p* = 0.04). The proportion of subjects who were rated as cooperative was comparable between the two groups. The findings of this study reinforce the notion that behavioral intervention should be employed as an efficacious strategy to equip subjects with ASDs with the requisite skills to undergo a dental examination.	-
Da Silva Moro et al. (2024) [45]	1. Control group—did not watch video before consultation 2. Test group—watched the video	Up to 5	Frankl Behavioral Scale; Mean service time of each visit; Mean number of visits to the dentist required to complete all steps (1–12) of oral physical examination and dental prophylaxis	The results demonstrated that the mean number of consultations in the intervention group was 1.5 (±1.53), while in the control group, it was 2 (±1.77) (*p* ≤ 0.05). The utilization of the video modeling technique has the potential to reduce the frequency of dental consultations in children with autism.	Up to five visits
Gandhi et al. (2024) [40]	1. VM 2. TSS	2	Plaque scores; Gingival scores; The effectiveness of the intervention was based on a 5-point Likert Scale	Significant improvements in plaque and gingival scores were observed for the VM (0.68 ± 0.20; 0.59 ± 0.15) and TSS (0.50 ± 0.11; 0.40 ± 0.10) groups at the post-intervention stage when compared to the pre-intervention visits. No statistically significant differences were observed in plaque or gingival scores between the VM and TSS groups. The VM group demonstrated encouraging outcomes in terms of caregivers’ perceptions regarding their children’s acceptance of oral hygiene practices.	4 weeks
Piraneh et al. (2023) [41]	1. Control group—Social story 2. Test group—VM	2	OHI-S; Oral health knowledge and attitude scores of the parents	The improvement in OHI-S was markedly greater in the intervention group. The use of video modeling based on modern technologies in an educational intervention for tooth brushing can result in a greater improvement in oral hygiene status than traditional social stories (standard education) in individuals with autism spectrum disorder (ASD).	4 weeks
Shalabi et al. (2022) [47]	1. VM 2. PECS	4	OHI-S; CI-s; DI-s	The VM group exhibited a statistically significant reduction in mean OHI-S scores in comparison to the PECS group over the follow-up period (*p* < 0.001). At the three-, six-, and 12-month marks, the mean differences in the OHI-S scores were 0.30, 0.58, and 0.57, respectively. For both groups, there was a moderate correlation between the severity of ASD and OHI-S scores at 12 months.	12 months
Stein Duker et al. (2023) [48]	1. RDE 2. SADE	2	Electrodermal activity; Tonic skin conductance level; Frequency per minute of nonspecific skin conductance responses; Children’s Dental Behavior Rating Scale	Children showed less stress during dental care in SADE than in RDE. There was a notable decrease in sympathetic activity and an increase in relaxation during SADE dental care. No significant differences were observed in the non-specific skin conductance responses. The frequency and duration of behavioral distress were significantly lower in the SADE group. There was a correlation between physiological stress and behavioral distress during the dental cleaning.	6 months

DVA—Dental Visual Aid, OH—Oral Hygiene, ASD—autism spectrum disorder, ASE Adaptive Sensory Environment, mYPAS—Modified Yale Preoperative Anxiety Scale, VN—Video Modeling, TSS—Tooth brushing social story, OHI-S—Simplified Oral Hygiene Index, PECS—Picture Exchange Communication System, DI-s—Simplified Debris Index, CI-s—Simplified Calculus Index, RDE—Regular Dental Environment, SADE—Sensory-Adapted Dental Environment.

**Table 6:** Summary of findings (SoF) and quality of evidence (GRADE).

Outcome	Number of Studies	Number of Patients	Study Design	Risk of Bias	Inconsistency	Indirectness	Imprecision	Publication Bias	Quality of Evidence	Importance
Oral Hygiene	Four	272	RCT	Low	Low	Low	Moderate	None detected	High	Critical
Anxiety and Stress	Two	88	RCT	Low	Moderate	Low	Moderate	None detected	Moderate	Critical
Behavioral Improvement	Five	237	RCT	Low	Low	Low	Moderate	None detected	High	Important

RCT—Randomized Controlled Trial.

## References

[1] Campisi L., Imran N., Nazeer A., Skokauskas N., Azeem M.W. (2018). Autism Spectrum Disorder. Br. Med. Bull..

[2] Loo C.Y., Graham R.M., Hughes C.V. (2009). Behaviour Guidance in Dental Treatment of Patients with Autism Spectrum Disorder. Int. J. Paediatr. Dent..

[3] Frye R.E. (2022). A Personalized Multidisciplinary Approach to Evaluating and Treating Autism Spectrum Disorder. J. Pers. Med..

[4] Klintwall L., Holm A., Eriksson M., Carlsson L.H., Olsson M.B., Hedvall Å., Gillberg C., Fernell E. (2011). Sensory Abnormalities in Autism. Res. Dev. Disabil..

[5] Than A., Patterson G., Cummings K.K., Jung J., Cakar M.E., Abbas L., Bookheimer S.Y., Dapretto M., Green S.A. (2024). Sensory Over-responsivity and Atypical Neural Responses to Socially Relevant Stimuli in Autism. Autism Res..

[6] Hsieh J.-J., Nagai Y., Kumagaya S., Ayaya S., Asada M. (2022). Atypical Auditory Perception Caused by Environmental Stimuli in Autism Spectrum Disorder: A Systematic Approach to the Evaluation of Self-Reports. Front. Psychiatry.

[7] Stein L.I., Polido J.C., Mailloux Z., Coleman G.G., Cermak S.A. (2011). Oral Care and Sensory Sensitivities in Children with Autism Spectrum Disorders. Spec. Care Dent..

[8] Lord C., Brugha T.S., Charman T., Cusack J., Dumas G., Frazier T., Jones E.J.H., Jones R.M., Pickles A., State M.W. (2020). Autism Spectrum Disorder. Nat. Rev. Dis. Primers.

[9] Salari N., Rasoulpoor S., Rasoulpoor S., Shohaimi S., Jafarpour S., Abdoli N., Khaledi-Paveh B., Mohammadi M. (2022). The Global Prevalence of Autism Spectrum Disorder: A Comprehensive Systematic Review and Meta-Analysis. Ital. J. Pediatr..

[10] Talantseva O.I., Romanova R.S., Shurdova E.M., Dolgorukova T.A., Sologub P.S., Titova O.S., Kleeva D.F., Grigorenko E.L. (2023). The Global Prevalence of Autism Spectrum Disorder: A Three-Level Meta-Analysis. Front. Psychiatry.

[11] Klein U., Nowak A.J. (1999). Characteristics of Patients with Autistic Disorder (AD) Presenting for Dental Treatment: A Survey and Chart Review. Spec. Care Dent..

[12] Limeres-Posse J., Castano-Novoa P., Abeleira-Pazos M., Ramos-Barbosa I. (2014). Behavioural Aspects of Patients with Autism Spectrum Disorders (ASD) That Affect Their Dental Management. Med. Oral.

[13] Gandhi R., Ruxmohan S., Puranik C.P. (2021). Association Between Autism Spectrum Disorder and Dental Anomalies of the Permanent Dentition. Pediatr. Dent..

[14] Suhaib F., Saeed A., Gul H., Kaleem M. (2019). Oral Assessment of Children with Autism Spectrum Disorder in Rawalpindi, Pakistan. Autism.

[15] Da Silva S.N., Gimenez T., Souza R.C., Mello-Moura A.C.V., Raggio D.P., Morimoto S., Lara J.S., Soares G.C., Tedesco T.K. (2017). Oral Health Status of Children and Young Adults with Autism Spectrum Disorders: Systematic Review and Meta-analysis. Int. J. Paediatr. Dent..

[16] Carli E., Pasini M., Pardossi F., Capotosti I., Narzisi A., Lardani L. (2022). Oral Health Preventive Program in Patients with Autism Spectrum Disorder. Children.

[17] Piraneh H., Gholami M., Sargeran K., Shamshiri A.R. (2022). Oral Health and Dental Caries Experience among Students Aged 7–15 Years Old with Autism Spectrum Disorders in Tehran, Iran. BMC Pediatr..

[18] Hariyani N., Oktarina, Shoaib L.A., Rohani M.M., Hanna K.M.B., Lee H. (2024). Caregivers’ Perceptions, Beliefs and Behavior Influence Dental Caries Experience in Children with Autism Spectrum Disorder: A Qualitative Study. Saudi Dent. J..

[19] Tsai S.-J., Hsu J.-W., Huang K.-L., Bai Y.-M., Su T.-P., Chen T.-J., Chen M.-H. (2023). Autism Spectrum Disorder and Periodontitis Risk. J. Am. Dent. Assoc..

[20] AlOtaibi A., Ben Shaber S., AlBatli A., AlGhamdi T., Murshid E. (2021). A Systematic Review of Population-Based Gingival Health Studies among Children and Adolescents with Autism Spectrum Disorder. Saudi Dent. J..

[21] Pimentel Júnior N.S., De Barros S.G., De Jesus Filho E., Vianna M.I.P., Santos C.M.L., Cangussu M.C.T. (2024). Oral Health-Care Practices and Dental Assistance Management Strategies for People with Autism Spectrum Disorder: An Integrative Literature Review. Autism.

[22] Alshatrat S.M., Al-Bakri I.A., Al-Omari W.M. (2020). Dental Service Utilization and Barriers to Dental Care for Individuals with Autism Spectrum Disorder in Jordan: A Case-Control Study. Int. J. Dent..

[23] Kenney M.K., Kogan M.D., Crall J.J. (2008). Parental Perceptions of Dental/Oral Health Among Children With and Without Special Health Care Needs. Ambul. Pediatr..

[24] Fallea A., Vetri L., L’Episcopo S., Bartolone M., Zingale M., Di Fatta E., d’Albenzio G., Buono S., Roccella M., Elia M. (2024). Oral Health and Quality of Life in People with Autism Spectrum Disorder. J. Clin. Med..

[25] Fallea A., Zuccarello R., Roccella M., Quatrosi G., Donadio S., Vetri L., Calì F. (2022). Sensory-Adapted Dental Environment for the Treatment of Patients with Autism Spectrum Disorder. Children.

[26] Niemczyk W., Balicz A., Lau K., Morawiec T., Kasperczyk J. (2024). Factors Influencing Peri-Extraction Anxiety: A Cross-Sectional Study. Dent. J..

[27] Popple B., Wall C., Flink L., Powell K., Discepolo K., Keck D., Mademtzi M., Volkmar F., Shic F. (2016). Brief Report: Remotely Delivered Video Modeling for Improving Oral Hygiene in Children with ASD: A Pilot Study. J. Autism Dev. Disord..

[28] Harris H.K., Weissman L., Friedlaender E.Y., Neumeyer A.M., Friedman A.J., Spence S.J., Rotman C., Krauss S., Broder-Fingert S., Weitzman C. (2024). Optimizing Care for Autistic Patients in Health Care Settings: A Scoping Review and Call to Action. Acad. Pediatr..

[29] Schardt C., Adams M.B., Owens T., Keitz S., Fontelo P. (2007). Utilization of the PICO Framework to Improve Searching PubMed for Clinical Questions. BMC Med. Inform. Decis. Mak..

[30] Page M.J., McKenzie J.E., Bossuyt P.M., Boutron I., Hoffmann T.C., Mulrow C.D., Shamseer L., Tetzlaff J.M., Akl E.A., Brennan S.E. (2021). The PRISMA 2020 Statement: An Updated Guideline for Reporting Systematic Reviews. BMJ.

[31] Watson P.F., Petrie A. (2010). Method Agreement Analysis: A Review of Correct Methodology. Theriogenology.

[32] Guyatt G.H., Oxman A.D., Vist G.E., Kunz R., Falck-Ytter Y., Alonso-Coello P., Schünemann H.J. (2008). GRADE: An Emerging Consensus on Rating Quality of Evidence and Strength of Recommendations. BMJ.

[33] Zink A.G., Molina E.C., Diniz M.B. (2018). Communication Application for Use During the First Dental Visit for Children and Adolescents with Autism Spectrum Disorders. Am. Acad. Pediatr. Dent..

[34] Nilchian F., Shakibaei F., Jarah Z.T. (2017). Evaluation of Visual Pedagogy in Dental Check-Ups and Preventive Practices Among 6–12-Year-Old Children with Autism. J. Autism Dev. Disord..

[35] Mah J.W., Tsang P. (2016). Visual Schedule System in Dental Care for Patients with Autism: A Pilot Study. J. Clin. Pediatr. Dent..

[36] Lowe O., Lindemann R. (1985). Assessment of the Autistic Patient’s Dental Needs and Ability to Undergo Dental Examination. ASDC J. Dent. Child..

[37] Isong I.A., Rao S.R., Holifield C., Iannuzzi D., Hanson E., Ware J., Nelson L.P. (2014). Addressing Dental Fear in Children with Autism Spectrum Disorders: A Randomized Controlled Pilot Study Using Electronic Screen Media. Clin. Pediatr..

[38] Cermak S.A., Stein Duker L.I., Williams M.E., Dawson M.E., Lane C.J., Polido J.C. (2015). Sensory Adapted Dental Environments to Enhance Oral Care for Children with Autism Spectrum Disorders: A Randomized Controlled Pilot Study. J. Autism Dev. Disord..

[39] Higgins J.P.T., Thomas J., Chandler J., Cumpston M., Li T., Page M.J., Welch V.A. (2024). Cochrane Handbook for Systematic Reviews of Interventions.

[40] Gandhi R., Jackson J., Puranik C.P. (2024). A Comparative Evaluation of Video Modeling and Social Stories for Improving Oral Hygiene in Children with Autism Spectrum Disorder: A Pilot Study. Spec. Care Dent..

[41] Piraneh H., Gholami M., Sargeran K., Shamshiri A.R. (2023). Social Story Based Toothbrushing Education Versus Video-Modeling Based Toothbrushing Training on Oral Hygiene Status Among Male Students Aged 7–15 Years Old with Autism Spectrum Disorders in Tehran, Iran: A Quasi-Randomized Controlled Trial. J. Autism Dev. Disord..

[42] Aljubour A., AbdElBaki M., El Meligy O., Al Jabri B., Sabbagh H. (2022). Effect of Culturally Adapted Dental Visual Aids on Oral Hygiene Status during Dental Visits in Children with Autism Spectrum Disorder: A Randomized Clinical Trial. Children.

[43] Aljubour A., AbdElBaki M., El Meligy O., Al-Jabri B., Sabbagh H. (2023). Effect of Culturally Adapted Dental Visual Aids on Anxiety Levels in Children with Autism Spectrum Disorder: A Randomized Clinical Trial. Children.

[44] Aljubour A.A., AbdElBaki M., El Meligy O., Al Jabri B., Sabbagh H. (2024). Culturally Adapted Dental Visual Aids Effect on Behavior Management during Dental Visits in Children with Autism Spectrum Disorder. J. Contemp. Dent. Pract..

[45] Da Silva Moro J., Rodrigues T.D., Kammer P.V., De Camargo A.R., Bolan M. (2024). Efficacy of the Video Modeling Technique as a Facilitator of Non-Invasive Dental Care in Autistic Children: Randomized Clinical Trial. J. Autism Dev. Disord..

[46] Cirio S., Salerno C., Mbanefo S., Oberti L., Paniura L., Campus G., Cagetti M.G. (2022). Use of Visual Pedagogy to Help Children with ASDs Facing the First Dental Examination: A Randomized Controlled Trial. Children.

[47] Shalabi M.A.S.A., Khattab N.M.A., Elheeny A.A.H. (2022). Picture Examination Communication System Versus Video Modelling in Improving Oral Hygiene of Children with Autism Spectrum Disorder: A Prospective Randomized Clinical Trial. Pediatr. Dent..

[48] Stein Duker L.I., Como D.H., Jolette C., Vigen C., Gong C.L., Williams M.E., Polido J.C., Floríndez-Cox L.I., Cermak S.A. (2023). Sensory Adaptations to Improve Physiological and Behavioral Distress During Dental Visits in Autistic Children: A Randomized Crossover Trial. JAMA Netw. Open.

[49] Shukla-Mehta S., Miller T., Callahan K.J. (2010). Evaluating the Effectiveness of Video Instruction on Social and Communication Skills Training for Children With Autism Spectrum Disorders: A Review of the Literature. Focus. Autism Other Dev. Disabl..

[50] Piccin S., Crippa A., Nobile M., Hardan A.Y., Brambilla P. (2018). Video Modeling for the Development of Personal Hygiene Skills in Youth with Autism Spectrum Disorder. Epidemiol. Psychiatr. Sci..

[51] Aljubour A., AbdElBaki M.A., El Meligy O., Al Jabri B., Sabbagh H. (2021). Effectiveness of Dental Visual Aids in Behavior Management of Children with Autism Spectrum Disorder: A Systematic Review. Child. Health Care.

[52] Murshid E.Z. (2017). Effectiveness of a Preparatory Aid in Facilitating Oral Assessment in a Group of Saudi Children with Autism Spectrum Disorders in Central Saudi Arabia. Saudi Med. J..

[53] Cagetti M., Mastroberardino S., Campus G., Olivari B., Faggioli R., Lenti C., Strohmenger L. (2015). Dental Care Protocol Based on Visual Supports for Children with Autism Spectrum Disorders. Med. Oral.

[54] Cermak S.A., Stein Duker L.I., Williams M.E., Lane C.J., Dawson M.E., Borreson A.E., Polido J.C. (2015). Feasibility of a Sensory-Adapted Dental Environment for Children With Autism. Am. J. Occup. Ther..

[55] Duker L.S., Polido J., Cermak S. (2021). Sensory Adapted Dental Environments to Enhance Oral Care for Children with Autism Spectrum Disorder. Pediatrics.

[56] Ismail A.F., Tengku Azmi T.M.A., Malek W.M.S.W.A., Mallineni S.K. (2021). The Effect of Multisensory-Adapted Dental Environment on Children’s Behavior toward Dental Treatment: A Systematic Review. J. Indian Soc. Pedod. Prev. Dent..

[57] Avery J.A., Ingeholm J.E., Wohltjen S., Collins M., Riddell C.D., Gotts S.J., Kenworthy L., Wallace G.L., Simmons W.K., Martin A. (2018). Neural Correlates of Taste Reactivity in Autism Spectrum Disorder. NeuroImage Clin..

[58] Zerman N., Zotti F., Chirumbolo S., Zangani A., Mauro G., Zoccante L. (2022). Insights on Dental Care Management and Prevention in Children with Autism Spectrum Disorder (ASD). What Is New?. Front. Oral Health.

[59] Al-Batayneh O.B., Nazer T.S., Khader Y.S., Owais A.I. (2020). Effectiveness of a Tooth-Brushing Programme Using the Picture Exchange Communication System (PECS) on Gingival Health of Children with Autism Spectrum Disorders. Eur. Arch. Paediatr. Dent..

[60] Zink A.G., Diniz M.B., Rodrigues Dos Santos M.T.B., Guaré R.O. (2016). Use of a Picture Exchange Communication System for Preventive Procedures in Individuals with Autism Spectrum Disorder: Pilot Study. Spec. Care Dent..

[61] Orellana L.M., Martínez-Sanchis S., Silvestre F.J. (2014). Training Adults and Children with an Autism Spectrum Disorder to Be Compliant with a Clinical Dental Assessment Using a TEACCH-Based Approach. J. Autism Dev. Disord..

[62] AlHumaid J., Tesini D., Finkelman M., Loo C.Y. (2016). Effectiveness of the D-TERMINED Program of Repetitive Tasking for Children with Autism Spectrum Disorder. J. Dent. Child..

[63] Rapin I., Tuchman R.F. (2008). Autism: Definition, Neurobiology, Screening, Diagnosis. Pediatr. Clin. N. Am..

[64] Jaber M.A. (2011). Dental Caries Experience, Oral Health Status and Treatment Needs of Dental Patients with Autism. J. Appl. Oral Sci..

[65] Muraru D., Ciuhodaru T., Iorga M. (2017). Providing dental care for children with autism spectrum disorders. Int. J. Med. Dent..

